# 

**Published:** 1884-11

**Authors:** 


Whole Number, CCLXX.

NOVEMBER, 1884.

Number 4, Volume XXIV.

### THE BUFFALO Medical and Surgical Journal.

LIBRARY

EDITORS:

THOS. LOTHROP, M. D., A. R. DAVIDSON, M. D.,

Published by the Medical Journal Association.

No. 5 WEST CHIPPEWA ST.

Entered at the Post-Office at Buffalo, N. Y., as Second-class Mail Matter.

